# The 100-Miles for QVOICES (Queer Voices in Oncology Igniting Change Through Empowered Stories of Survivorship) Campaign: Overview of the Benefits and Costs of Crowdfunding as a Funding Mechanism for Research

**DOI:** 10.2196/80079

**Published:** 2026-02-26

**Authors:** Viktor Clark, Milena E Insalaco, Charles K Kamen, Lauren V Ghazal

**Affiliations:** 1 Division of Supportive Care in Cancer School of Medicine University of Rochester Rochester, NY United States; 2 Wilmot Cancer Center University of Rochester Rochester, NY United States; 3 School of Nursing University of Rochester Rochester, NY United States

**Keywords:** crowdfunding, early-stage investigators, LGBTQ+, cancer, survivorship

## Abstract

In today’s unstable funding climate, alternative research funding mechanisms are essential. Investigators are facing novel barriers to acquiring funding for their research. Early-stage investigators, in particular those who are beginning their careers in research, are especially vulnerable to these funding disruptions. Currently, finding alternative funding is especially relevant for scientists studying historically and intentionally excluded communities, such as lesbian, gay, bisexual, transgender, queer, or other sexual and/or gender expansive (LGBTQ+) populations, as many investigators have had their LGBTQ+ grants revoked by the Trump administration. Crowdfunding for research studies is a potential funding avenue that has grown in popularity, with more than US $12 million raised since 2012. This Viewpoint highlights crowdfunding as a potential funding model for LGBTQ+ cancer research, specifically to support the collection of preliminary data for predoctoral students and early-stage investigators. We describe the benefits and challenges of using crowdfunding as the sole funding mechanism for a mixed methods observational study among LGBTQ+ survivors of cancer. Despite challenges, crowdfunding can fund rigorous research on health disparities, notably in historically and intentionally excluded communities.

## Background

### Predoctoral Students and Early-Stage Investigators

Predoctoral students are currently enrolled in a doctoral program and eligible to conduct research [1], whereas early-stage investigators are defined as researchers in the early years (typically within 10 years) of their terminal degree who have not yet received a substantial independent research award (eg, R00 and R01) [2]. As of 2022, there were more than 250,000 full-time doctoral students in the fields of science, engineering, and health [3]; more than 55,000 doctoral degrees were awarded, more than 34,000 medical students were matched, and more than 62,000 postdoctoral trainees began their careers in the United States, representing a combined generation of early-stage investigators and predoctoral students [4]. As required by many independent research awards, predoctoral students and early-stage investigators must acquire preliminary data to achieve a successful funding application [5]. However, there are limited government-supported funding mechanisms that provide resources to develop such preliminary data (eg, small R03s, R21s, and R15s) [6], and these have recently become even more restricted for research investigating historically and intentionally excluded communities [7]. Determining innovative strategies for collecting preliminary data among historically and intentionally excluded communities is necessary to ensure that researchers focusing on these disparities can progress in their careers and that the health disparities faced by these communities continue to be studied to work toward amelioration.

### Crowdfunding

Crowdfunding (ie, the raising of money, capital, or other resources from a large number of people to support a project or venture) [8] is an innovative strategy that has been used to assist in funding for-profit business ventures, nonprofit social entrepreneurship projects, and even to offset medical expenses [9]. Since 2012, crowdfunding has grown, even within the scientific community, especially on platforms such as Experiment.com, which has been used to help fund more than 3000 research projects and raise more than US $12 million [10]. Crowdfunding has emerged as a notable phenomenon that researchers are examining to identify which types of projects are best supported by this financing model [11]. The growth of crowdfunding has been particularly notable among students and early-stage investigators who may require alternative funding pathways beyond traditional government-based models [12]. Crowdfunding may be particularly salient in light of the current funding crisis as a result of numerous executive orders signed by President Donald Trump [13].

### Current Status of Research for Lesbian, Gay, Bisexual, Transgender, Queer, or Other Sexual and/or Gender Expansive Communities

Even before the executive orders implemented by President Trump, funding for research focused on some specific minoritized communities has been bleak, specifically among lesbian, gay, bisexual, transgender, queer, or other sexual and/or gender expansive (LGBTQ+) research efforts. Between 1989 and 2014, the National Institutes of Health (NIH) allocated 0.1% of its total budget toward LGBTQ+ research that was not related to HIV [14]. In 2020, this increased to 0.8%, when NIH funded 500 non–HIV-related projects pertaining to LGBTQ+ individuals (US $284,051,683) [15]. In 2015, NIH developed the Office of Sexual and Gender Minority Research, which helped support leading efforts to collect sexual orientation and gender identity data to determine the extent of health disparities between LGBTQ+ and cisgender heterosexual individuals [16]. With the support of the Office of Sexual and Gender Minority Research, longitudinal studies have been conducted that have revealed substantive disparities in health care for LGBTQ+ individuals. Examples of these disparities include higher rates of drug, alcohol, and tobacco use; greater mental health concerns, especially regarding aging; and a higher risk of several cancers (eg, skin, cervical, and anal) among LGBTQ+ individuals compared with cisgender heterosexual individuals [17,18]. In early 2025, the Office of Sexual and Gender Minority Research was dismantled through executive orders issued by President Trump [19]. Furthermore, in these executive orders, LGBTQ+ research has been deemed superfluous, leading to significant gaps in governmental funding [20]. As of 2025, nearly 10% (1 in 10) of the US adult population identifies as LGBTQ+ [21]. This proportion is even higher among younger generations, with nearly 30% (1 in 3) identifying as LGBTQ+ [22]. Consequently, health care systems are now tasked with providing care to LGBTQ+ individuals, yet many remain ill-equipped to do so [23]. However, disparities faced by LGBTQ+ people and gaps in knowledge regarding their experiences, particularly across the cancer control continuum, persist [24].

### Viewpoint Aim

As these disparities persist and government funding becomes more restricted, the need for alternative funding for LGBTQ+ cancer research remains a high priority. In this Viewpoint, we highlight crowdfunding as a potential funding model for LGBTQ+ cancer research as an alternative to government-funded research for the collection of preliminary data, particularly for predoctoral students and early-stage investigators. We describe how crowdfunding was used for the QVOICES (Queer Voices in Oncology Igniting Change through Empowered stories of Survivorship) study, a mixed methods observational study among LGBTQ+ survivors of cancer.

## Study Overview

### Crowdfunding for QVOICES

**Figure 1:**
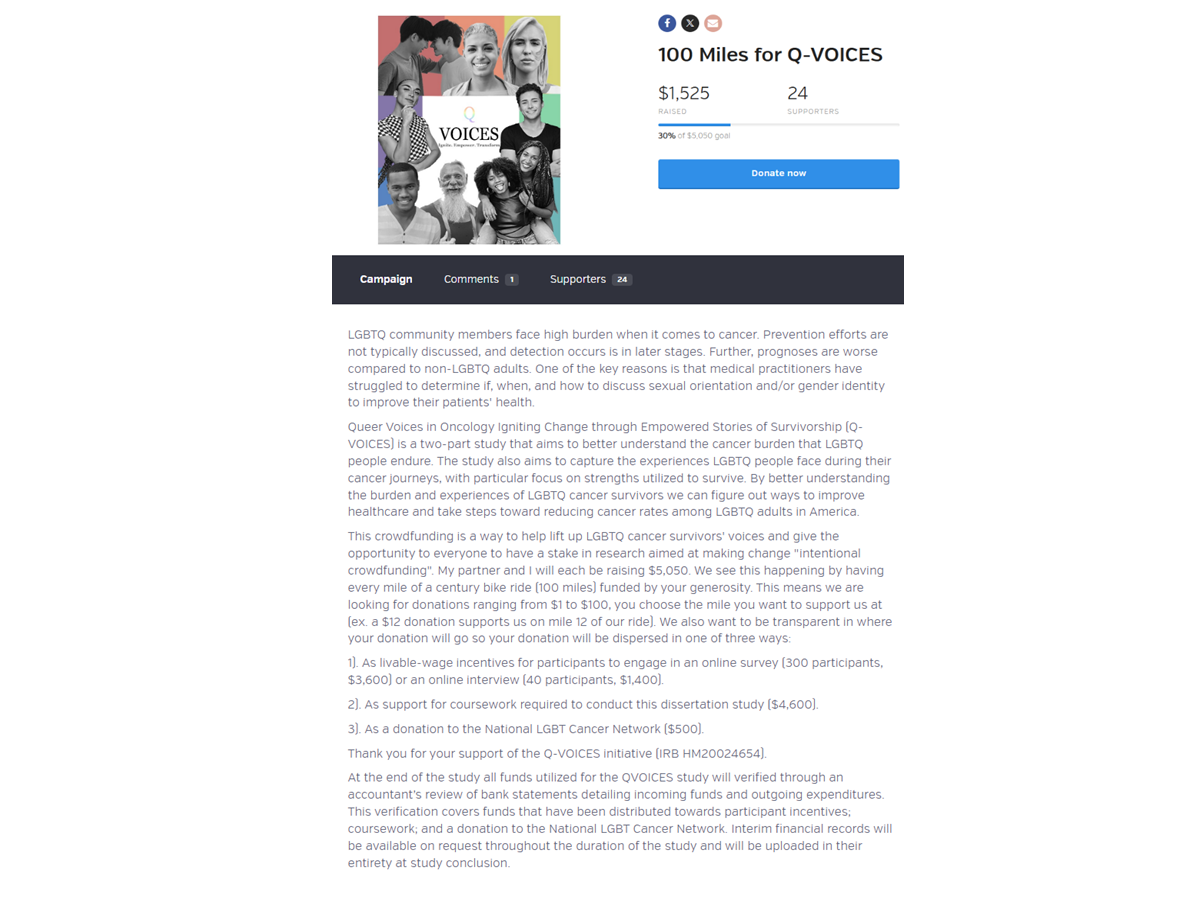
QVOICES (Queer Voices in Oncology Igniting Change through Empowered stories of Survivorship) website.

In 2022, the QVOICES study was launched [25]. QVOICES was a mixed methods dissertation led by the first author (VC) and used crowdfunding to provide livable wage incentives for participants in the study. The QVOICES crowdfunding campaign was titled “100 Miles for QVOICES.” The first author (VC) engaged in a 100-mile (160-km) bike ride in support of this campaign. Donors to the crowdfunding campaign were encouraged to make donations ranging from US $1 to US $100. The campaign was shared across social media platforms (ie, Instagram [Meta Platforms, Inc] and Facebook [Meta Platforms, Inc]) and through email. Both social media ads and emails provided links that directed potential donors to a main crowdfunding webpage hosted on BetterWorld, a fundraising and crowdfunding platform focused mainly on nonprofit, charitable, and social impact initiatives [26]. BetterWorld was chosen due to its elimination of hosting fees, unlike other crowdfunding platforms such as GoFundMe or Kickstarter [26]. The main crowdfunding webpage had details about the QVOICES project and a self-created video that gave an overview of how donors’ funds would be used (Figure 1).

Funds were split into 3 intentions. First, funds were allocated to participant incentives. Second, funds were applied to offset study costs. Third, any remaining funds were donated to the National LGBT Cancer Network [27].

**Figure 2:**
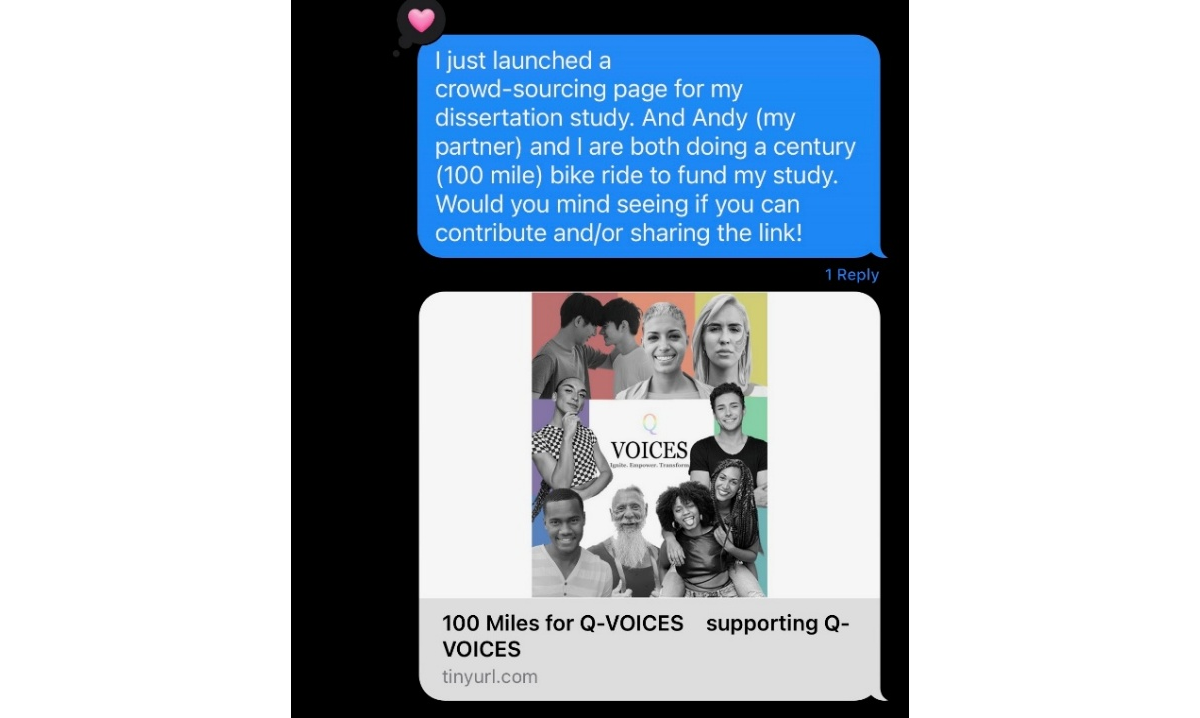
100-Miles for QVOICES (Queer Voices in Oncology Igniting Change through Empowered stories of Survivorship) texts.

**Figure 3:**
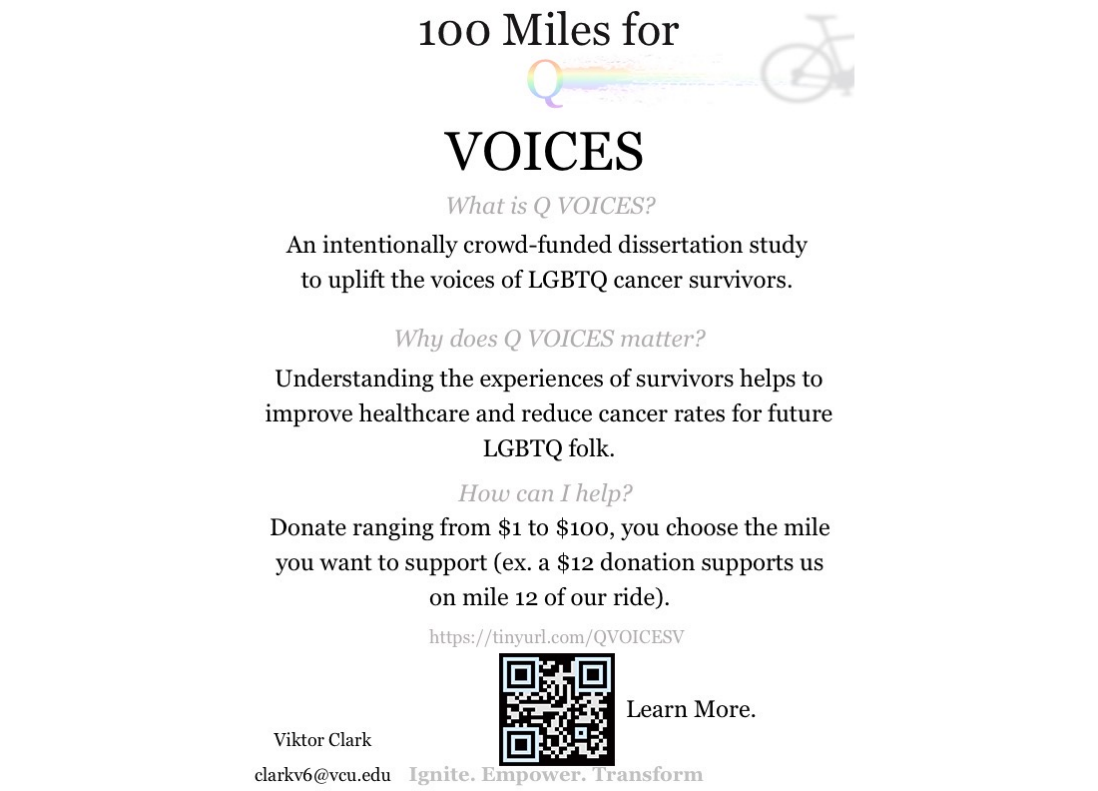
100-Miles for QVOICES (Queer Voices in Oncology Igniting Change through Empowered stories of Survivorship) electronic flyer.

**Figure 4:**
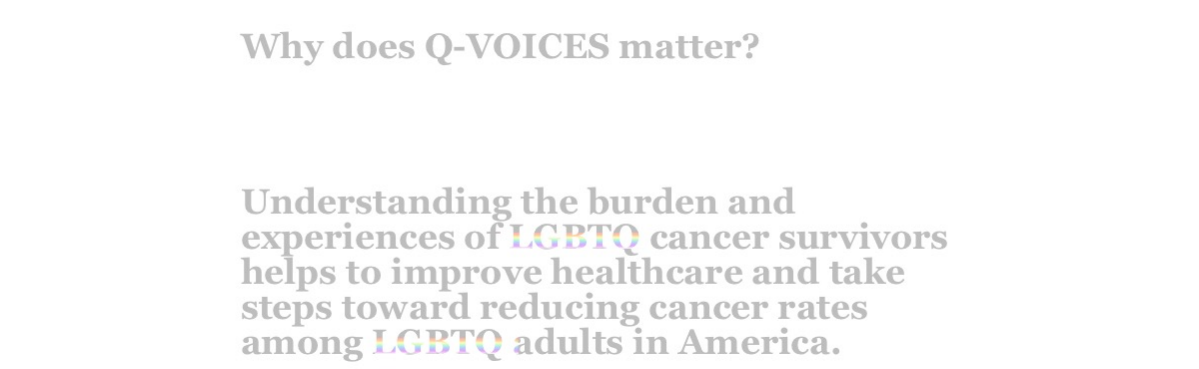
100-Miles for QVOICES (Queer Voices in Oncology Igniting Change through Empowered stories of Survivorship) Instagram carousel.

To reach key stakeholders, crowdfunding campaign materials were distributed in several ways. First, texts and emails were sent to the social networks of the first author (VC), including friends and family (Figure 2). Second, emails with an electronic flyer (Figure 3) were sent to (1) diversity, equity, and inclusion offices; (2) faculty engaged in LGBTQ+ research efforts, identified through faculty biographies, publication histories, and abstracts; (3) National Cancer Institute–designated cancer centers determined to be LGBTQ+ inclusive, identified using the National LGBT Cancer Network “LGBT Welcoming Treatment Providers” tool [28]; and (4) LGBTQ+ affinity groups at various universities. At each National Cancer Institute–designated cancer center, faculty listed as LGBT-friendly providers by the National LGBT Cancer Network tool were specifically contacted. Within the LGBTQ+ affinity groups, the president or vice president was contacted if their information was available; if not, the general information email was used. Finally, the campaign was also posted in LGBTQ+ Facebook groups and on Instagram using Instagram carousels (Figure 4), with numerous hashtags (eg, #QVOICES, #crowdfunding, #LGBTQ, #cancer, #research, #donate, #ignite, #empower, and #transform).

The 100 Miles for QVOICES crowdfunding campaign ran from March 1, 2022, to June 30, 2022. Approximately 500 emails and texts were distributed, and Facebook ads were run daily during this period. An estimated US $150 was spent on Facebook ads. For marketing efforts, Instagram stories were also created every other day on a QVOICES-specific account between March 1, 2022, and June 30, 2022. QVOICES was approved by the Virginia Commonwealth University institutional review board (IRB) as an exempt study for all participant engagement. Funding initiatives through crowdsourcing were outside the scope of IRB jurisdiction.

### Crowdfunding Results and Benefits

#### Overview

Crowdfunding efforts resulted in a total of US $2218 from 26 donors. Of these donations, the majority (n*=*24, 92%) were acquired through text messages from friends and family or through word-of-mouth referrals from friends and family. Two donations were received from faculty at institutions engaged in LGBTQ+ research.

#### Benefits

A crowdfunding approach to the QVOICES project led to several benefits. First, researchers were able to engage with community stakeholders and other scientists [29]. Second, branding around QVOICES established a means for all project-related materials to be interconnected [30]. Third, there was validation that LGBTQ+ focused cancer research was a priority, as evidenced by funding support [31]. Finally, maintaining autonomy in all funding decisions allowed for the use of innovative and attractive incentives (eg, participants were able to select their own gift cards [1 of 75 different types]) as compensation for engagement in the study [32].

## Lessons Learned

### Engaging With Community Stakeholders and Scientists

Throughout the distribution of crowdfunding materials, community stakeholders and scientists came together to help spread the word about the 100 Miles for QVOICES campaign. LGBTQ+ community members and scientists were often not direct sources of donations but instead served as champions by sharing information with allies who ultimately contributed. This was an interesting phenomenon and may be explained by LGBTQ+ community members historically being tasked with raising funds for their own initiatives and a new mentality shift toward collaborating with those in privileged positions to make change for marginalized communities [33].

Regarding engaging with scientists, past research has argued that those with postgraduate degrees have been found to be one of the lowest groups to contribute to crowdfunding efforts [29]. However, although their contributions to crowdfunding have been low, academic scientists over the past decade have been more accepting of using crowdfunding as a practical way to offset funding shortages and continue research initiatives [34].

### Ethics of Crowdfunding

As noted, QVOICES being funded through crowdfunding efforts meant that the materials we disseminated to donors were not subject to IRB oversight. However, we carefully maneuvered how to engage with our donors and demonstrate tangible results from their contributions. On our donation page, we had a disclaimer stating that donating to the QVOICES project did not provide donors with rights to individual participant data; rather, the data would be reported as summative data in accordance with the IRB.

It was also important for us to be as transparent as possible with the use of funds that we were raising; therefore, we had detailed information on fund allocation laid out in a three-item financial budget: (1) livable wage participant incentives (US $8 per 15-minute survey and US $40 per hour interview), (2) research costs (ie, transcription software), and (3) a donation to the National LGBT Cancer Network if goal of US $5000 was met. We also informed donors that QVOICES had specific branding and that, in accordance with this branding, donors could search for and find any publications or conference proceedings connected to the QVOICES study. Donors’ names, should they want them to appear in any disseminated materials, would be included on all manuscripts and conference proceedings, similar to that of a funding agency. All these efforts were to enhance the ethics of keeping the robustness and high quality of data collection at a peak, to keep the confidentiality of our participants safe, and to have our donors stay engaged and recognize that we were devoted to using their funds in a meaningful way.

### Branding

Due to the increasing number of crowdfunding efforts, branding has become even more essential. Branding is defined as a means to differentiate oneself from competitors [35] and can include logos, messaging, and calls to action [36]. Successful branding has been determined to be instrumental in crowdfunding decision-making [37]. During the process of developing the 100-Miles QVOICES campaign, branding was an essential target in our development.

**Figure 5:**
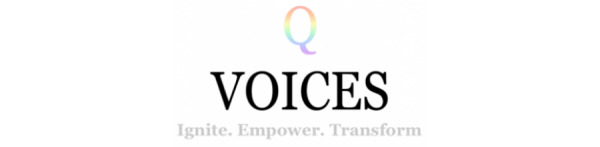
QVOICES logo.

This was achieved through several means. First, QVOICES developed a distinct logo that was applied to all materials distributed for the campaign and the study (Figure 5). The pastel rainbow coloring of the “Q” in the logo intentionally established the LGBTQ+ community as the target audience. We also used a simple 3-word tagline, “Ignite. Empower. Transform,” which summarized our intention across all QVOICES efforts to use LGBTQ+ voices to *ignite* change, *empower* individuals, and *transform* cancer outcomes for these communities. Now, at the dissemination phase of QVOICES, this logo is placed on all posters, oral presentations, and manuscripts to recognize the crowdfunding donors and research participants who similarly supported the brand and research efforts of QVOICES.

### Validation and Prioritization of LGBTQ+ Cancer Research Among Donors

One of the most important aspects of crowdfunding is that financial support validates a crowd’s belief in an effort [38]. We were able to achieve somewhat of a local validation. Our initial goal was US $5000, and we were successful in funding US $2218 (44.4%). We also had a <5% of response rate, resulting in 26 individual donations. Among these donors, LGBTQ+ cancer research was seen as a priority. This was reflected in our study, requesting donations ranging from US $1 to $100 to represent the 100 miles that would be biked throughout the campaign and achieving an average of US $88 (SD US $68.68) per donation.

### Financial Autonomy

Finally, due to our funding resulting from crowdfunding, there were many aspects of the government-required financial process that we were able to avoid. One such process was the collection of social security numbers. Due to our community of interest (LGBTQ+ individuals), many participants were concerned about providing their social security numbers; therefore, reducing this barrier to participation was essential. In addition, often incentives need to be distributed at the conclusion of interviews or a few days later due to the processing of funds through systems. However, due to having autonomy in our funds, we were able to distribute our interview incentives at the beginning of the interview, allowing participants the autonomy to discontinue their participation at any point without penalty. We believe this approach led to more engagement, retention, and transparency throughout the interviews.

## Barriers to Crowdfunding

Although there were many benefits to crowdfunding, there were also some costs that came with using crowdfunding, both in our marketing stream of the 100-Miles for QVOICES campaign and in using crowdfunding as our sole source of funding.

### Mistrust or Stigma of the LGBTQ+ Topic Area

Because our campaign was run through the site BetterWorld, which is a newer platform and not as popular as Kickstarter or GoFundMe, this may have impacted donors’ trust in the campaign. Additionally, because the majority of emails sent were “cold” emails (ie, initial contact made with the individual by our study team) and social media posts were cast to a large network, acquiring trust that we were going to use donations toward the promised efforts may have been difficult to obtain. Furthermore, because the emails were sent cold, many administrators, faculty, affinity officers, social media users, and text recipients were unfamiliar with our principal investigator (VC), which may have also caused some hesitation in donating toward the 100-Miles for QVOICES efforts.

Additionally, a complex stigma has been established for LGBTQ+ academicians regarding whether to engage in or even endorse LGBTQ+ research [39]. This complexity has only grown due to the politically hostile environment created by various executive orders under the Trump presidency [13]. QVOICES data were collected from June 2020 to January 2021 (during the first Trump presidency). Within this time, more than 150 anti-LGBTQ+ bills were introduced into state legislation [40]. This politically hostile environment may have further dissuaded people from donating due to concerns regarding their academic standings, even if they were interested in supporting the findings of the QVOICES dissertation work. Future studies should examine how trust in the campaign, trust in the sponsor of the campaign, and trust in the project topic impact intent to donate and conversion to actual donation from key stakeholders.

### Lack of Sense of Urgency

In our campaign materials distributed through emails, texts, Facebook ads, and Instagram posts, we included (1) a blurb with a hyperlink to the BetterWorld crowdfunding site; (2) a video describing both the QVOICES study and the 100-mile campaign, with the website to the BetterWorld crowdfunding site included at the end; and (3) a PDF flyer for the 100-mile campaign with a call-to-action for how donors could get involved with a clickable hyperlink to the BetterWorld crowdfunding site. We felt that providing all 3 mediums maximized the potential for donations.

However, a more iterative and interactive modality may have resulted in better crowdfunding donation reactions [37]. This may have included developing Instagram posts with open comments, sending materials out in iterative waves (eg, first with blurbs about the study, then a flyer, and then a video) to keep the crowdfunding materials accessible to potential donors, and using daily updates and discussions through the “BetterWorld” campaign page. Overall, while we had many well-developed marketing materials, the implementation of these materials could have been improved.

Additionally, providing an option for participants to opt out of iterative marketing materials, with a reason (eg, not interested in the project or have received too many funding requests about the topic area), may help to establish if saturation among key stakeholders on the topic area has been reached and whether an expansion of stakeholders is necessary.

### Nuance

Crowdfunding has typically been used to fund business ventures and nonprofit social entrepreneurship projects and even to offset medical expenses [9]. Due to the nuance of crowdfunding being used within the research sector, this may have created some hesitation to engage in donation. According to the theory of Diffusion of Innovations [41], often with innovations, only 2.5% of individuals are seen as innovators or the first individuals to adopt an innovation, which is consistent with our low rate of return, as indicated by individuals who actually donated to our campaign.

If crowdfunding is to remain a permanent means of funding research, this rate should grow to approximately 13.4% of individuals, representing early adopters [41], followed by another 34% as the early majority, another 34% as the late majority [41], and finally 16% representing individuals who are the last to adopt crowdfunding as a reasonable means to fund research [41]. In alignment with the theory of Diffusion of Innovations, our goal of US $5000 was not met, and we had a <5% return rate. However, we see the 100-Miles for QVOICES campaign as the cutting edge of this innovation curve and anticipate crowdfunding to become more widely adopted as funding options shift.

### Future Directions

From the 100-Miles for QVOICES campaign and the QVOICES study, we have learned many fundamental lessons to carry out other successful crowdfunding campaigns for research in the future. One of the biggest takeaways from this campaign was that when given the opportunity, donors—if interested and if barriers to donation are overcome—will rise to support the efforts of researchers, when they trust the effort, know that the research will be carried out, and know that their funds will be used in an effective and transparent way.

Our biggest lesson learned is that having a brand is only one step of the solution toward a successful campaign. Additionally, ensuring that the brand is backed by a strong marketing strategy, that there are large audiences to pivot toward, and that techniques exist to overcome barriers such as stigma are all essential to reaching the overarching goal of a campaign—particularly when conducting LGBTQ+ research. Future directions should apply these strategies to (1) various funding initiatives to enable comparative metrics to determine if topic area matters and (2) expand funding initiatives beyond solely participant incentives (eg, study materials, personnel, and salary support).

### Conclusions

Crowdfunding can be a functional method for funding research that may need minimal support from nontraditional avenues (eg, outside of government funding or foundational grants), such as that of preliminary data collection among early-stage investigators. As government funding becomes more constrained and foundational grants become more saturated, crowdfunding provides an additional alternative for financial support [42]. Through the 100-Miles of QVOICES campaign, we have seen a glimpse of success that a crowdfunding campaign can bring in ensuring that high-quality research can be conducted and disseminated. Although crowdfunding can come with various challenges, with thoughtful planning, branding, marketing, and support, the possibilities of using crowdfunding as an innovative strategy to conduct research addressing health disparities is achievable.

## Abbreviations

IRB: institutional review board
LGBTQ+: lesbian, gay, bisexual, transgender, queer, or other sexual and/or gender expansive
NIH: National Institutes of Health
QVOICES: Queer Voices in Oncology Igniting Change through Empowered stories of Survivorship
